# Comparison of health measures between survey self-reports and electronic health records among Millennium Cohort Study participants receiving Veterans Health Administration care

**DOI:** 10.1186/s12874-025-02529-x

**Published:** 2025-03-27

**Authors:** Felicia R. Carey, Elaine Y. Hu, Nicole Stamas, Amber Seelig, Lynne Liu, Aaron Schneiderman, William Culpepper, Rudolph P. Rull, Edward J. Boyko, for the Millennium Cohort Study Team, Anna Baccetti, Jennifer N Belding, Satbir K. Boparai, Marvin A. Jr. Brown, Nathan Carnes, Sheila F. Castañeda, Rebecca A. Consigli, Toni Rose Geronimo-Hara, Judith Harbertson, Lauren Jackson, Isabel G. Jacobson, Claire K. Kolaja, Cynthia A. LeardMann, Crystal L. Lewis, David Moreno Ignacio, Erin L. Richard, Anna C. Rivera, Neika Sharifian, Beverly D. Sheppard, Daniel W. Trone, Javier Villalobos, Jennifer L. Walstrom, Yunnuo Zhu

**Affiliations:** 1 Deployment Health Research Department, Naval Health Research Center, 140 Sylvester Road, San Diego, CA 92106-3521 USA; 2 Seattle Epidemiologic Research and Information Center, Department of Veterans Affairs, VA Puget Sound Healthcare System, Seattle, WA USA; 3 Health Outcomes Military Exposures, Veterans Health Administration, Department of Veterans Affairs, Washington, DC USA

**Keywords:** Diagnosis, Self-report, Agreement, Longitudinal research, Electronic health records

## Abstract

**Background:**

Surveys are a useful tool for eliciting self-reported health information, but the accuracy of such information may vary. We examined the agreement between self-reported health information and medical record data among 116,288 military service members and veterans enrolled in a longitudinal cohort.

**Methods:**

Millennium Cohort Study participants who separated from service and registered for health care in the Veterans Health Administration (VHA) by September 18, 2020, were eligible for inclusion. Baseline and follow-up survey responses (2001–2016) about 39 medical conditions, health behaviors, height, and weight were compared with analogous information from VHA and military medical records. Medical record diagnoses were classified as one qualifying ICD code in any diagnostic position between October 1, 1999, and September 18, 2020. Additional analyses were restricted to medical record diagnoses occurring before survey self-report and using specific diagnostic criteria (two outpatient or one inpatient ICD code). Positive, negative, and overall (Youden’s *J*) agreement was calculated for categorical outcomes; Bland–Altman plots were examined for continuous measures.

**Results:**

Among 116,288 participants, 71.8% self-reported a diagnosed medical condition. Negative agreement between self-reported and VHA medical record diagnoses was > 90% for most (80%) conditions, but positive agreement was lower (6.4% to 56.3%). Mental health conditions were more frequently recorded in medical records, while acute conditions (e.g., bladder infections) were self-reported at a higher frequency. Positive agreement was lower when analyses were restricted to medical record diagnoses occurring prior to survey self-report. Specific diagnostic criteria resulted in higher overall agreement.

**Conclusions:**

While negative agreement between self-reported and medical record diagnoses was high in this population, positive and overall agreement were not strong and varied considerably by health condition. Though the limitations of survey-reported health conditions should be considered, using multiple data sources to examine health outcomes in this population may have utility for research, clinical planning, or public health interventions.

**Supplementary Information:**

The online version contains supplementary material available at 10.1186/s12874-025-02529-x.

## Background

Understanding the level of concordance between conditions found in health records and patient self-reports is crucial for determining accurate disease prevalence and assessing patient–provider communication [1, 2]. Medical records are frequently utilized in epidemiological research as the criterion standard of patient health information, but they are subject to limitations such as non-standardized coding, fragmentation of information from multiple facilities, delayed recording, and subjective reporting by the provider [3–7]. Self-reports may provide a more complete health history but are similarly affected by limitations due to recall bias, patient health literacy, and other individual factors [1, 5, 8].

Several studies have investigated the agreement between medical records and patient self-reported conditions with inconsistent results, often varying by condition type, age, health status, sample size, and study design. The Millennium Cohort Study previously compared medical conditions reported in self-administered surveys collected from 37,798 U.S. service members and veterans and linked objective health records maintained in the Military Health System Data Repository (MDR) [1]. The study found near-perfect negative agreement and moderate positive agreement between these two sources, suggesting that self-report may be sufficient to exclude a history of conditions not otherwise documented in the health record [1]. Another large cross-sectional study comparing health surveys with health records found a higher average prevalence of conditions in self-reports and noted that the survey had higher sensitivity in identifying symptomatic conditions, such as chronic allergies, neck and back pain, and arthritis [9]. Other studies have reported a similar overrepresentation of symptom-based conditions among self-reports, including migraine headaches [1, 9], prostatitis [1, 7], urinary tract infections [7], fractures [10], and osteoarthritis [1, 6, 9, 11]. Several studies have provided evidence for high agreement (kappa ≥ 0.70) among chronic conditions, such as diabetes, hypertension, and cancer, and conditions with well-defined diagnostic criteria, including myocardial infarction and stroke [1, 2, 4, 6, 8, 10, 12]. In addition to varied agreement across conditions, multiple studies have reported significant differences in concordance by age [2–4, 10], number of comorbidities [2, 3, 6], and frequency of health care utilization [2, 10]. A longer archival period (or longer enrollment period) also appeared to be associated with increased average concordance by allowing more time for conditions to be documented in the medical record [1, 2, 8]. Two studies used multiple strategies to ascertain cases from health records that included augmentation with lab tests, pharmacy records, and physician notes [11, 12]. None of these studies, however, compared self-report and health records agreement across different case ascertainment criteria and time periods.

Reliability of available patient health information is particularly important among military populations, where force readiness depends on the health of service members. Among veterans, a higher frequency of multiple physical and mental health conditions may signal a need for improved health care delivery to address complex comorbidities [13]. Through linkage with triennial survey data from the U.S. Department of Defense (DoD) Millennium Cohort Study and the Veterans Health Administration (VHA) of the Department of Veterans Affairs, the current study will augment previous research in this population to compare up to 20 years of self-reported survey data from multiple survey administration cycles with VHA medical record diagnoses among participants who utilized VHA for care [1].

The objective of this analysis was to compare diagnoses of 39 conditions of interest in medical records (VHA inpatient, outpatient, and purchased care; United States Renal Data System [USRDS]; and MDR) with diagnoses reported on the Millennium Cohort Study survey. The aims were to determine (1) the agreement between positive responses for the 39 conditions of interest on the Millennium Cohort Study survey with a corresponding diagnosis in medical records, (2) the agreement between negative responses for the 39 conditions of interest on the Millennium Cohort Study survey without a corresponding diagnosis in medical records, and (3) a measure of overall concordance between the two sources.

## Methods

### Study population

The Millennium Cohort Study is the DoD’s largest prospective cohort study of U.S military service members and veterans. Initiated in 2001 in response to the National Defense Authorization Act for Fiscal Year 1999, which directed the DoD to establish a longitudinal study examining the health impacts of deployments and associated exposures, the study has collected self-report, medical record, and administrative data on military service and health outcomes spanning up to 21 years [14–17]. Detailed descriptions of the Study and its findings from the past two decades are available elsewhere [14, 15]. The initial panel and each of three subsequent panels of Millennium Cohort Study participants were selected as a random sample from Defense Manpower Data Center (DMDC) rosters of military personnel from all service branches (Army, Navy, Coast Guard, Air Force, and Marine Corps) and Reserve/National Guard personnel, weighted to oversample for specific populations [14, 16, 17]. While all panels oversampled for female service members, Panel 1 (enrolled 2001–2003) oversampled for Reserve/National Guard members and personnel with previous deployment history [16], Panels 2 (2004–2005) and 3 (2007–2008) oversampled for Marine Corps personnel, and Panel 4 (2011–2013) oversampled for married service members. This analysis examined data from participants from these first four enrollment panels.

Millennium Cohort Study baseline questionnaires and triennial follow-up surveys include validated survey instruments to measure military-related exposures and participant health outcomes, including physician-diagnosed conditions, self-reported symptoms, mental health assessment, physical and functional status, alcohol use, tobacco use, sleep patterns, life experiences, and occupational exposures [14–17]. Survey responses are augmented with data from DMDC personnel files to capture baseline participant characteristics, such as sex, age, education level, marital status, race and ethnicity, pay grade, deployment experience, service branch, length of service, and military occupation [17], and linked with MDR records to capture medical encounters occurring within the Military Health System (MHS). Participants continue to participate in the study even after separating from service and records are also linked to VHA records to capture medical encounters occurring within multiple healthcare systems. For the purposes of this study, veteran characteristics were assessed using the most recent VHA record and included whether participants had a service-connected disability and VHA health care utilization, which was categorized based on average number of VHA encounters per year, with “regular users” defined as those with at least 1 encounter per year on average, “irregular users” as those with fewer than 1 encounter per year on average but at least 1 encounter for the period of observation, and “high frequency users” as those with more than 1 encounter per year every year during the period of observation. The period of observation for determining VHA health care utilization was measured from the first VHA encounter on record to the last encounter date available for each participant prior to September 18, 2020.

### Defining cases in the medical record

International Classification of Diseases, Ninth Revision (ICD-9) and Tenth Revision (ICD-10), and Current Procedural Terminology (CPT) codes for each of the 39 conditions of interest were selected by a clinician and verified by the research team (see Additional File 1). CPT and ICD procedure codes were additionally used to identify kidney failure requiring dialysis. Relevant diagnostic codes were extracted from VHA files (inpatient, outpatient, and purchased care encounters), USRDS data (for kidney failure requiring dialysis), and MDR medical records (inpatient and outpatient encounters) for the time period beginning at the start of fiscal year 1999 and ending on September 18, 2020. For determining the presence of a condition in medical records, sensitive criteria defined a positive case as the presence of any relevant diagnostic code (ICD-9, ICD-10, or CPT) located in any diagnostic position found in either inpatient or outpatient medical records for the 39 conditions of interest. Additional analyses utilized specific criteria, which defined a positive case in the medical record as the presence of at least two outpatient codes or one inpatient diagnostic code located in any diagnostic position. For example, if only one diagnostic code for a given condition was identified in outpatient medical records only, a participant would be flagged as a positive case for that condition based on sensitive criteria but would not be identified as a case using specific criteria. For both sensitive and specific criteria, the first encounter date or admission date when a given condition was noted defined the diagnosis date.

### Defining cases in Millennium Cohort Study surveys

Self-reported cases were assessed via a survey item asking, “Has a doctor or other health professional told you that you have any of the following conditions?” ever at baseline, or in the past 3 years at follow-up. If participants responded “Yes” to a specific condition at any survey assessment between 2001 and 2016, they were classified as a positive case at all subsequent waves, with the diagnosis date corresponding to the survey date at first self-report. Otherwise, if they did not respond “Yes” at any wave and responded “No” at any wave, they were classified as a negative case. Conditions with missing responses at all available survey waves, which account for < 0.02% of all participants across the 39 conditions, were set to missing.

### Defining temporal sequence

For primary analyses, no restrictions were placed on the temporal sequence such that diagnostic codes in medical records could appear at any time in relation to survey self-report. This approach recognizes the clinical circumstance in which a patient may be informed of a likely diagnosis with confirmation of that diagnosis not appearing in the medical record until a future time. Additional analyses restricted diagnostic codes to those appearing *prior* to the date of survey self-report to better inform the interpretation of concordance between a self-reported condition and a diagnosis confirmed in the medical record. Codes indicating personal history of a condition were not utilized in additional analyses investigating temporality because these codes lack a date of diagnosis.

### Health behaviors and body measurements

Objective height and weight measurements from VHA vital signs medical records were compared with survey-reported height and weight, with weight measurements considered if taken within 1 year of a corresponding survey. For instances in which more than one weight was taken within 1 year of a survey, the weight recorded closest to the date of survey was used. When multiple height measurements were available, the mode was used for both medical records and survey responses. For instances in which all available height measurements were different, the first height recorded was used. Weights < 80 lb or > 500 lb and heights < 48 inches or > 95 inches were omitted from analysis as extreme values.

Problem drinking was assessed on the Millennium Cohort Study survey using 5 items from the Patient Health Questionnaire (PHQ) assessing risky alcohol use behaviors, with probable problem drinking defined as positive endorsement of at least 1 of the 5 items (e.g., driving a car after drinking too much) in the last 12 months [18]. Self-reported alcohol use in the medical record was taken from VHA health factors data and assessed using the Alcohol Use Disorders Identification Test-Concise (AUDIT-C) alcohol dependence screening tool, with scores of 3 or more for women or 4 or more for men indicating alcohol misuse [19]. The AUDIT-C assessment closest in time prior to a completed survey date was compared with the survey PHQ responses. If more than one AUDIT-C alcohol assessment was completed on the same day for a participant, the highest score was used.

Participants were classified as ever smokers via survey responses if they reported at any survey wave that they had smoked 100 cigarettes or more in their lifetime. Smoking was assessed in medical records via VHA health factors data, using the smoking assessments recorded closest to a corresponding survey date.

### Statistical analysis

Demographic, military, and veteran characteristics were examined by enrollment panel and among those who self-reported one or more conditions. The prevalence of the 39 conditions of interest in survey records, VHA medical records only, and combined VHA-MDR medical records were examined, as well as the agreement between these sources. Positive and negative agreement were calculated for the comparisons of self-reported conditions with those diagnosed in medical records [20, 21]. For the purposes of this study, we calculated positive agreement as 2a/[N + (a—d)] and negative agreement as 2d/[N—(a—d)], where “a” represents true positives for the condition, “d” represents true negatives for the condition, and N represents the total number of individuals. Youden’s *J* statistic was used to measure overall concordance between the medical record and survey responses [22] because this measure accounts for both sensitivity and specificity and is therefore independent of the prevalence of conditions of interest [22, 23]. Positive and negative agreement and Youden’s *J *were also calculated to compare health behaviors (smoking and alcohol use) between survey self-reports and medical records. Bland–Altman plots were used to examine the agreement between self-reported and medical record measurements of height and weight and evaluate bias between the mean differences of these measurements [24].

The main analyses compared survey self-reported conditions with medical record conditions classified using sensitive criteria and occurring at any time in relation to survey completion from VHA medical records only or from combined VHA-MDR medical records. Three additional variations of the main analyses were conducted: (1) with medical record diagnoses classified using specific criteria and occurring at any time in relation to survey completion, (2) with medical record diagnoses classified using sensitive criteria and temporally restricted to those occurring prior to survey completion, and (3) with medical record diagnoses classified using specific criteria and temporally restricted to those occurring prior to survey completion. The sensitivity and specificity of self-report in detecting medical record diagnoses by each case attainment strategy are reported in Additional File 2. Additional supplemental analyses investigated whether concordance of survey and medical record diagnosis varied by VHA user frequency. SAS software, version 9.4 (Cary, NC) was used for all analyses.

## Results

### Participant characteristics

Of the 133,163 Millenium Cohort Study participants who had separated from service during the study period, 116,288 participants (or 87.3%) were identified in VHA records. Demographic, military, and veteran characteristics among these 116,288 participants are listed in Table 1. Among participants, 71.8% self-reported ever being told by a health professional that they had one or more of the 39 medical conditions on the survey. Specifically, a higher proportion of those who were female, in older age groups, married or separated/divorced, or had a service-connected disability, self-reported one or more medical conditions. Overall, 57.4% of participants were classified as high frequency VHA users.

**Table 1:** Characteristics of Millennium Cohort Study participants, by panel and number of conditions (*N*=116,288)

	**Panel 1**		**Panel 2**		**Panel 3**		**Panel 4**	
**Characteristic**^a^	All participants	Reported ≥1 conditions^b^	All participants	Reported ≥1 conditions^b^	All participants	Reported ≥1 conditions^b^	All participants	Reported ≥1 conditions^b^
	*N*=48,491	*n*=40,301	*N*=18,012	*n*=12,050	*N*=24,004	*n*=14,790	*N*=25,781	*n*=16,333
	n (%)	n (%)	n (%)	n (%)	n (%)	n (%)	n (%)	n (%)
**Demographic characteristics**								
**Sex**								
Male	35,439 (73.1)	28,742 (71.3)	10,870 (60.4)	6647 (55.2)	15,274 (63.6)	8491 (57.4)	18,435 (71.5)	10,908 (66.8)
Female	13,052 (26.9)	11,559 (28.7)	7142 (39.7)	5403 (44.8)	8730 (36.4)	6299 (42.6)	7346 (28.5)	5425 (33.2)
**Age (years)**								
17–24	6653 (13.7)	4657 (11.6)	11,413 (63.4)	7285 (60.5)	15,263 (63.6)	8982 (60.7)	9438 (36.6)	5647 (34.6)
25–34	17,025 (35.1)	13,475 (33.4)	5447 (30.2)	3854 (32.0)	7779 (32.4)	5074 (34.3)	13,963 (54.2)	8935 (54.7)
35–44	17,970 (37.1)	15,741 (39.1)	999 (5.6)	781 (6.5)	908 (3.8)	692 (4.7)	2067 (8.0)	1502 (9.2)
45+	6843 (14.1)	6428 (16.0)	153 (0.9)	130 (1.1)	54 (0.2)	42 (0.3)	313 (1.2)	249 (1.5)
**Education level**								
Some high school	135 (0.3)	103 (0.3)	83 (0.5)	41 (0.3)	58 (0.2)	32 (0.2)	54 (0.2)	24 (0.2)
High school graduate	7807 (16.1)	6020 (14.9)	5570 (30.9)	3499 (29.0)	7146 (29.8)	4039 (27.3)	5798 (22.5)	3589 (22.0)
Some college	19,942 (41.1)	16,494 (40.9)	8547 (47.5)	5843 (48.5)	11,800 (49.2)	7462 (50.5)	12,581 (48.8)	8164 (50.0)
College graduate	15,100 (31.1)	12,854 (31.9)	3198 (17.8)	2212 (18.4)	4380 (18.3)	2809 (19.0)	6244 (24.2)	3840 (23.5)
Advanced degree	5506 (11.4)	4829 (12.0)	612 (3.4)	455 (3.8)	619 (2.6)	448 (3.0)	1103 (4.3)	716 (4.4)
**Marital status**								
Never married	9310 (19.2)	6931 (17.2)	9458 (52.5)	5955 (49.4)	11,874 (49.5)	6785 (45.9)	7981 (31.0)	4488 (27.5)
Married	31,978 (66.0)	27,134 (67.3)	6881 (38.2)	4819 (40.0)	9780 (40.7)	6270 (42.4)	14,067 (54.6)	9136 (55.9)
Separated, divorced, or widowed	7203 (14.9)	6236 (15.5)	1672 (9.3)	1276 (10.6)	2350 (9.8)	1735 (11.7)	3733 (14.5)	2709 (16.6)
**Race and ethnicity**								
Asian/Pacific Islander	1327 (2.7)	1073 (2.7)	781 (4.3)	450 (3.7)	1212 (5.1)	676 (4.6)	1205 (4.7)	670 (4.1)
Black non-Hispanic	8366 (17.3)	6951 (17.3)	2321 (12.9)	1560 (13.0)	2869 (12.0)	1705 (11.5)	2859 (11.1)	1769 (10.8)
Hispanic	3357 (6.9)	2650 (6.6)	1916 (10.6)	1252 (10.4)	2059 (8.6)	1182 (8.0)	2536 (9.8)	1502 (9.2)
Other	1191 (2.5)	999 (2.5)	338 (1.9)	222 (1.8)	704 (2.9)	422 (2.9)	747 (2.9)	488 (3.0)
White non-Hispanic	34,229 (70.6)	28,609 (71.0)	12,640 (70.2)	8556 (71.0)	17,160 (71.5)	10,805 (73.1)	18,434 (71.5)	11,904 (72.9)
**Military characteristics**								
**Military pay grade**								
Enlisted	38,932 (80.3)	32,155 (79.8)	16,530 (91.8)	11,019 (91.4)	22,209 (92.5)	13,659 (92.4)	23,278 (90.3)	14,879 (91.1)
Officer	9559 (19.7)	8146 (20.2)	1482 (8.2)	1031 (8.6)	1795 (7.5)	1131 (7.7)	2503 (9.7)	1454 (8.9)
**Deployment experience**								
Nondeployed	21,688 (44.7)	18,836 (46.7)	4794 (26.6)	3443 (28.6)	5539 (23.1)	3610 (24.4)	5548 (21.5)	3593 (22.0)
Deployed	26,803 (55.3)	21,465 (53.3)	13,218 (73.4)	8607 (71.4)	18,465 (76.9)	11,180 (75.6)	20,233 (78.5)	12,740 (78.0)
**Service branch**								
Army	24,611 (50.8)	20,491 (50.8)	9747 (54.1)	6683 (55.5)	10,182 (42.4)	6842 (46.3)	13,725 (53.2)	9373 (57.4)
Navy/Coast Guard	8045 (16.6)	6748 (16.7)	2740 (15.2)	1884 (15.6)	3867 (16.1)	2332 (15.8)	3808 (14.8)	2235 (13.7)
Marine Corps	2451 (5.1)	1906 (4.7)	1658 (9.2)	1113 (9.2)	4501 (18.8)	2649 (17.9)	3018 (11.7)	1957 (12.0)
Air Force	13,384 (27.6)	11,156 (27.7)	3867 (21.5)	2370 (19.7)	5454 (22.7)	2967 (20.1)	5230 (20.3)	2768 (17.0)
**Length of service** (years)^‡^								
0–5	2792 (5.8)	2082 (5.2)	6320 (35.1)	4478 (37.2)	7856 (32.7)	5220 (35.3)	7427 (28.8)	5289 (32.4)
6–10	4334 (8.9)	3293 (8.2)	5292 (29.4)	3549 (29.5)	8837 (36.8)	5454 (36.9)	15,597 (60.5)	9444 (57.8)
11–15	2996 (6.2)	2400 (6.0)	5115 (28.4)	3095 (25.7)	6948 (29.0)	3889 (26.3)	2489 (9.7)	1423 (8.7)
16+	38,366 (79.1)	32,523 (80.7)	1285 (7.1)	928 (7.7)	363 (1.5)	227 (1.5)	268 (1.0)	177 (1.1)
**Occupational category**								
Combat specialist	9071 (18.7)	7244 (18.0)	2999 (16.7)	1983 (16.5)	3938 (16.4)	2424 (16.4)	4243 (16.5)	2781 (17.0)
Other occupations	39,420 (81.3)	33,057 (82.0)	15,013 (83.4)	10,067 (83.5)	20,066 (83.6)	12,366 (83.6)	21,538 (83.5)	13,552 (83.0)
**Veteran characteristics**								
**VHA use**								
Irregular users	4586 (9.5)	3756 (9.3)	1130 (6.3)	649 (5.4)	1179 (4.9)	658 (4.5)	1062 (4.1)	567 (3.5)
Regular users	17,667 (36.4)	15,043 (37.3)	7455 (41.4)	5147 (42.7)	8680 (36.2)	5585 (37.8)	7754 (30.1)	5144 (31.5)
High frequency users	26,237 (54.1)	21,501 (53.4)	9426 (52.3)	6253 (51.9)	14,145 (58.9)	8547 (57.8)	16,965 (65.8)	10,622 (65.0)
**Service-connected disability**								
Yes	38,054 (78.5)	32,671 (81.1)	12,408 (68.9)	8976 (74.5)	16,878 (70.3)	11,410 (77.2)	17,723 (68.7)	12,541 (76.8)
No	9985 (20.6)	7311 (18.1)	5491 (30.5)	3010 (25.0)	6911 (28.8)	3281 (22.2)	7712 (29.9)	3640 (22.3)
Unknown	452 (0.9)	319 (0.8)	113 (0.6)	64 (0.5)	215 (0.9)	99 (0.7)	346 (1.3)	152 (0.9)

*Note*: For Veterans Health Administration (VHA) use, irregular users are defined as individuals with less than 1 encounter/year on average but at least 1 encounter within the period of observation; regular users are defined as individuals with at least 1 encounter/year on average; high frequency users are defined as more than 1 encounter/year every year during the period of observation

^a^Demographic and military characteristics are measured at Millennium Cohort Study baseline. Veteran characteristics are taken from the most recent VHA record

^b^Participant self-reported 1 or more conditions on any Millennium Cohort Study survey

^‡^Length of service as of August 31, 2016

### Prevalence of medical conditions

The most prevalent condition identified by self-report, VHA records only, and VHA-MDR records was depression (26.5%, 39.5%, and 51.2% respectively), followed by migraine headaches (23.4%) and tinnitus (22.0%) for self-report surveys; posttraumatic stress disorder (PTSD; 30.3%) and sleep apnea (26.3%) for VHA records only; and sinusitis (41.0%) and sleep apnea (37.5%) for VHA-MDR records (Table 2). Mental health conditions, such as depression and PTSD, and endocrine/metabolic conditions, such as thyroid conditions and diabetes, were identified more often in medical records than in survey self-reports. Conditions associated with changing symptom severity, such as chronic fatigue syndrome, rheumatoid arthritis, and stomach/duodenal/peptic ulcer, were self-reported on surveys at a higher prevalence than what appeared in medical records. Most other conditions had comparable prevalence estimates between surveys and medical records or were slightly higher for surveys.

**Table 2:** Prevalence and agreement between self-report and medical record conditions, sensitive, at any time criterion (*N*=116,288)

	**Self-report**	**VHA records only**				**Combined VHA-MDR records**			
**Condition**	n (%)	n (%)	Positive agreement	Negative agreement	Youden’s *J*	n (%)	Positive agreement	Negative agreement	Youden’s *J*
**Diseases & Disorders of the Nervous System**									
Multiple sclerosis	887 (0.8)	476 (0.4)	31.5%	99.6%	0.45	775 (0.7)	31.5%	99.5%	0.33
Migraine headaches	27,209 (23.4)	18,916 (16.3)	50.4%	87.7%	0.45	27,541 (23.7)	59.6%	87.6%	0.47
Neuropathy	8553 (7.4)	10,263 (8.8)	27.2%	93.6%	0.19	23,775 (20.4)	30.1%	88.7%	0.16
Seizures	1868 (1.6)	2251 (1.9)	31.2%	98.8%	0.27	4212 (3.6)	31.2%	98.2%	0.22
Stroke	1091 (0.9)	1795 (1.5)	20.4%	99.0%	0.16	4099 (3.5)	19.0%	98.2%	0.11
Sleep apnea	18,414 (15.8)	30,561 (26.3)	46.4%	85.7%	0.29	43,663 (37.5)	48.6%	81.3%	0.30
**Diseases & Disorders of the Sense Organs**									
Significant hearing loss	20,006 (17.2)	23,426 (20.1)	46.6%	87.7%	0.33	36,055 (31.0)	48.7%	83.7%	0.30
Tinnitus	25,621 (22.0)	23,679 (20.4)	44.2%	85.0%	0.30	31,587 (27.2)	51.1%	84.0%	0.33
**Diseases & Disorders of the Respiratory System**									
Asthma	10,844 (9.3)	9890 (8.5)	47.9%	94.9%	0.45	16,295 (14.0)	50.2%	93.4%	0.38
Chronic bronchitis	7442 (6.4)	4753 (4.1)	16.1%	95.4%	0.15	21,022 (18.1)	22.7%	89.2%	0.11
Emphysema	1112 (1.0)	3508 (3.0)	13.1%	98.2%	0.08	5777 (5.0)	12.3%	97.3%	0.07
Sinusitis	23,333 (20.1)	15,412 (13.3)	30.2%	86.0%	0.21	47,672 (41.0)	44.1%	75.4%	0.22
**Diseases & Disorders of the Circulatory System**									
Hypertension	25,200 (21.7)	27,274 (23.5)	56.3%	87.3%	0.43	40,485 (34.8)	61.0%	84.6%	0.43
Coronary heart disease	1865 (1.6)	2405 (2.1)	30.5%	98.7%	0.26	6032 (5.2)	28.6%	97.5%	0.18
Angina	7300 (6.3)	2802 (2.4)	15.8%	96.2%	0.23	5718 (4.9)	24.4%	95.5%	0.23
Heart attack	1421 (1.2)	947 (0.8)	22.6%	99.2%	0.27	2111 (1.8)	33.9%	99.0%	0.28
Any other heart condition	9192 (7.9)	10,724 (9.2)	24.1%	92.9%	0.16	23,656 (20.3)	29.9%	88.5%	0.16
**Diseases & Disorders of the Digestive System**									
Stomach, duodenal, or peptic ulcer	6220 (5.4)	1182 (1.0)	10.9%	97.1%	0.29	2981 (2.6)	22.7%	96.8%	0.30
Ulcerative colitis or proctitis	1620 (1.4)	834 (0.7)	23.1%	99.2%	0.33	1448 (1.2)	28.5%	99.0%	0.29
Crohn’s disease	815 (0.7)	535 (0.5)	31.0%	99.6%	0.39	1086 (0.9)	30.4%	99.4%	0.26
**Diseases & Disorders of the Hepatobiliary System & Pancreas**									
Hepatitis B	1146 (1.0)	279 (0.2)	15.2%	99.5%	0.38	11,403 (9.8)	4.5%	94.6%	0.02
Hepatitis C	939 (0.8)	791 (0.7)	27.2%	99.5%	0.29	2524 (2.2)	15.7%	98.7%	0.10
Any other hepatitis	1411(1.2)	595 (0.5)	6.4%	99.2%	0.10	4170 (3.6)	7.7%	97.7%	0.04
Cirrhosis	719 (0.6)	474 (0.4)	7.9%	99.5%	0.09	726 (0.6)	10.0%	99.4%	0.09
Gallstones	4029 (3.5)	1911 (1.6)	14.3%	97.8%	0.19	5160 (4.4)	35.4%	97.3%	0.29
Pancreatitis	1160 (1.0)	770 (0.7)	16.5%	99.3%	0.20	1714 (1.5)	27.3%	99.1%	0.22
**Diseases & Disorders of the Musculoskeletal System & Connective Tissue**									
Rheumatoid arthritis	6675 (5.7)	1046 (0.9)	10.7%	96.9%	0.34	2186 (1.9)	17.9%	96.7%	0.31
Lupus	811 (0.7)	407 (0.4)	27.9%	99.6%	0.41	765 (0.7)	31.2%	99.5%	0.32
**Endocrine, Nutritional & Metabolic Diseases & Disorders**									
Thyroid condition other than cancer	5989 (5.2)	8769 (7.5)	45.8%	96.3%	0.36	15,476 (13.3)	44.7%	94.4%	0.30
Diabetes or sugar diabetes	5164 (4.4)	9315 (8.0)	42.3%	96.2%	0.31	14,592 (12.5)	41.0%	94.5%	0.27
**Diseases & Disorders of the Kidney & Urinary Tract**									
Bladder infection	11,798 (10.2)	1587 (1.4)	8.8%	94.4%	0.27	8592 (7.4)	30.6%	93.3%	0.28
Kidney failure requiring dialysis	587 (0.5)	249 (0.2)	11.5%	99.7%	0.19	318 (0.3)	11.3%	99.7%	0.16
**Disorders of Blood and Blood Forming Organs**									
Anemia	9261 (8.0)	9900 (8.5)	28.6%	93.6%	0.22	20,601 (17.7)	35.5%	90.5%	0.22
**Mental and Behavioral Disorders**									
Manic depressive disorder	3280 (2.8)	7850 (6.8)	23.3%	96.1%	0.15	10,466 (9.0)	22.9%	95.2%	0.13
Schizophrenia or psychosis	1150 (1.0)	2435 (2.1)	17.9%	98.7%	0.12	3942 (3.4)	15.2%	98.1%	0.09
Depression	30,780 (26.5)	45,946 (39.5)	55.8%	78.3%	0.33	59,504 (51.2)	58.3%	73.5%	0.36
Posttraumatic stress disorder	19,970 (17.2)	35,189 (30.3)	54.0%	85.7%	0.36	38,385 (33.0)	55.0%	84.9%	0.37
**Other Conditions**									
Cancer	4802 (4.1)	5255 (4.5)	35.5%	97.1%	0.31	10,697 (9.2)	42.9%	95.9%	0.30
Chronic fatigue syndrome	3865 (3.3)	1695 (1.5)	9.1%	97.8%	0.12	2357 (2.0)	10.1%	97.5%	0.10

*MDR* Military Health System Data Repository, *VHA* Veterans Health Administration

### Agreement between self-reported medical conditions and medical records

Overall, the positive agreement between self-report and VHA records only was low, ranging from 6.4% for any other hepatitis to 56.3% for hypertension (Table 2). Negative agreement across the 39 conditions ranged from 78.3% for depression to 99.7% for kidney failure. Concordance between self-report and VHA records ranged from slight (e.g., Youden *J* = 0.09 for cirrhosis) to moderate (e.g., Youden *J* = 0.45 for multiple sclerosis, migraine headaches, and asthma). When combined VHA-MDR records were examined, positive agreement across all conditions was comparable or higher (with the exception of hepatitis B and hepatitis C), negative agreement was comparable or lower, and concordance was comparable or lower (with the exception of gallstones).

The prevalence of, and agreement between, self-reported conditions and VHA records using varying case ascertainment strategies and by VHA user frequency (irregular, regular, or high) were examined. Comparing specific criteria and sensitive criteria for medical record diagnoses prior to the survey date resulted in higher concordance as assessed by Youden’s *J* for all diagnoses, but no consistent patterns were observed with respect to the magnitude of positive or negative agreement (see Additional File 3). Comparison of diagnoses occurring ever versus prior to survey date using specific criteria resulted in a lower Youden’s *J* but a higher positive agreement for nearly all diagnoses. No consistent pattern of differences across diagnoses was seen for positive agreement or Youden’s *J* comparing high and regular users of VHA health care (see Additional File 4). Irregular users of VHA health care had lower levels of positive agreement for most diagnoses compared with regular or high users.

Sensitivity analyses (Additional File 5) examined the prevalence of depression in VHA only and combined VHA-MDR records when ICD codes relevant to Unspecified Depressive Disorder were excluded. In excluding these codes, the agreement between self-reported conditions and VHA records remained consistent with the results reported in Table 2.

### Prevalence and agreement of health behaviors

The prevalence of ever smoking and problem drinking (Table 3) was higher for both in VHA records (61.4% and 43.8%, respectively) than in self-reported records (50.8% and 27.1%, respectively). Positive agreement between self-reported and VHA records was 83.2% for ever smoking and 49.2% for problem drinking, while negative agreement was 78.5% for ever smoking and 72.1% for problem drinking. Concordance was substantial for ever smoking (Youden *J* = 0.65) and fair for problem drinking (Youden *J* = 0.23).

**Table 3:** Prevalence and agreement of smoking and alcohol use between self-report and VHA records

Condition	Self-report^a^	VHA records^b^	Measures of agreement		
	n (%)	n (%)	Positive agreement	Negative agreement	Youden’s *J*
**Smoking**					
Ever smoker	42,178 (50.8)	50,960 (61.4)	83.2%	78.5%	0.65
**Alcohol use**					
Problem drinking	21,200 (27.1)	34,278 (43.8)	49.2%	72.1%	0.23

*AUDIT-C* Alcohol Use Identification Test-Concise, *PHQ* Patient Health Questionnaire, *VHA* Veterans Health Administration

^a^The Millennium Cohort Survey utilizes the PHQ to measure problem drinking

^b^VHA records utilize the AUDIT-C screening tool for alcohol dependence

### Height and weight

**Fig. 1:**
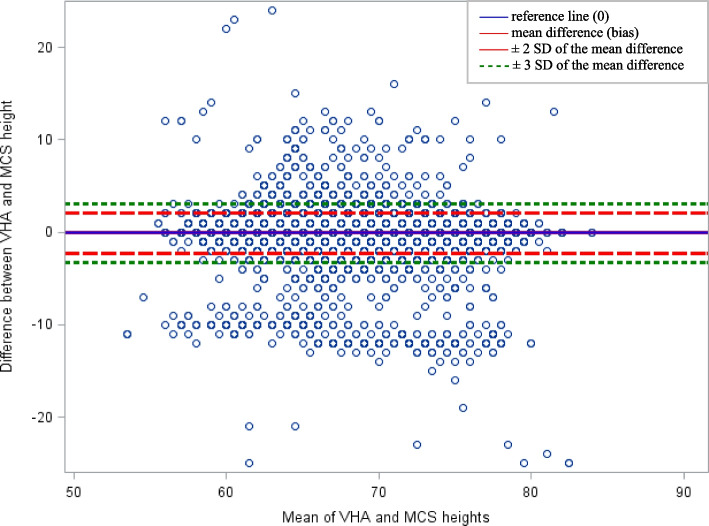
Bland–Altman plot comparing heights self-reported on the Millennium Cohort Study (MCS) survey and in Veterans Health Administration (VHA) medical records. SD, standard deviation

**Fig. 2:**
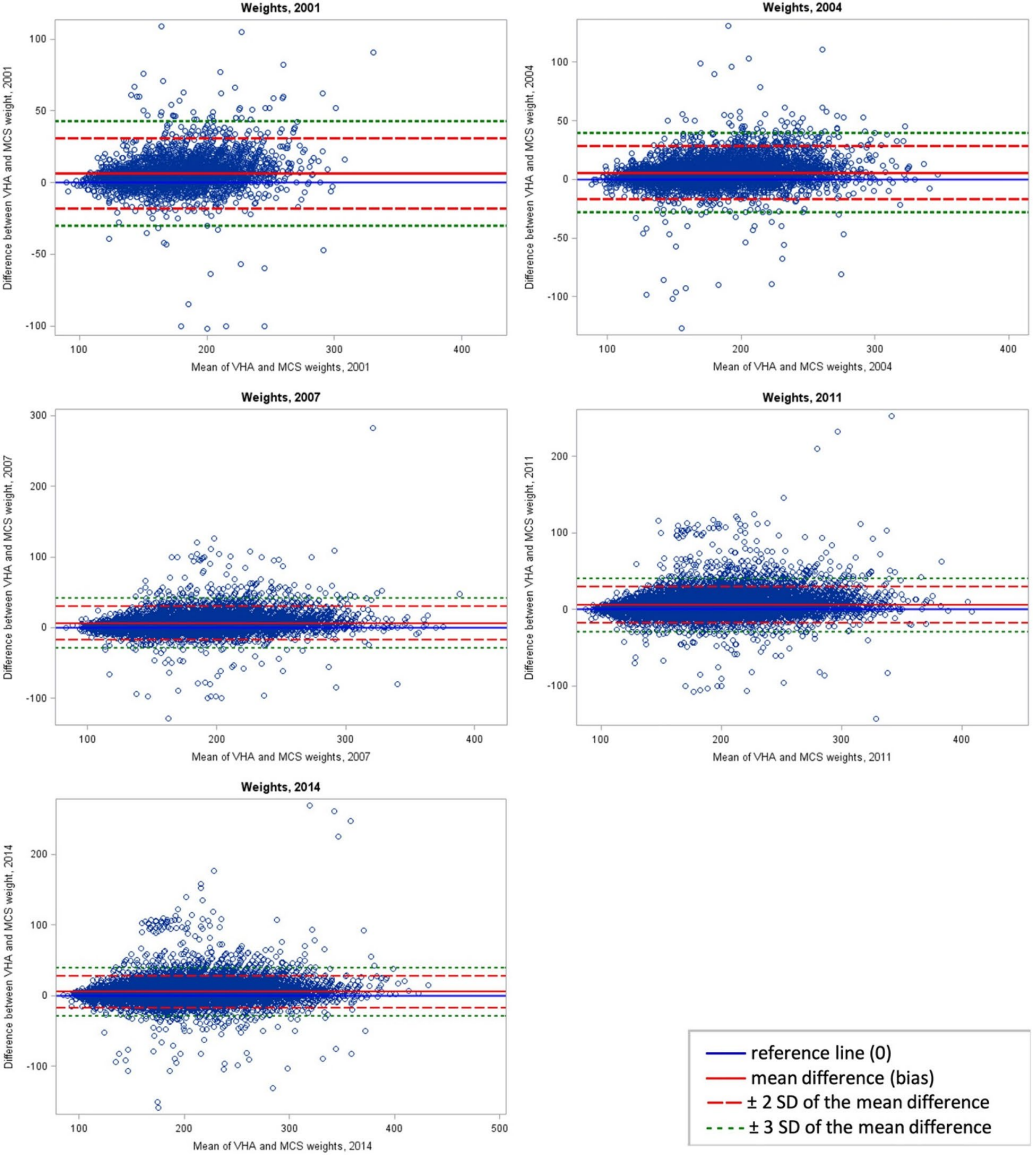
Bland–Altman plot comparing weights self-reported on the Millennium Cohort Study (MCS) survey and in Veterans Health Administration (VHA) medical records. SD, standard deviation

Agreement between heights is presented in a Bland–Altman plot (Fig. 1). The mean difference in height between VHA and self-reported records was −0.12 inches (SD = 1.07); 1.4% of paired measurements were more than 2 standard deviations from the mean difference. The reference line representing zero mean difference between both methods was within the confidence lines representing 2 standard deviations from the mean, suggesting no difference between the measures. Greater data clustering below the lower confidence line versus the upper confidence line suggests a skew in the data toward heights being higher in self-reported records than in VHA records. Agreement between weights by survey year is presented in Fig. 2. The mean difference in weight was 6.44 lb (SD = 12.21) at the 2001 survey point, 5.68 lb (SD = 11.31) at 2004, 5.99 lb (SD = 11.77) at 2007, 5.58 lb (SD = 11.67) at 2011, and 4.88 lb (SD = 11.28) at 2014. The proportion of paired measurements more than 2 standard deviations from the mean difference was 4.3% at 2001, 4.1% at 2004, 3.9% at 2007, 4.0% at 2011, and 3.6% at 2014. The reference lines representing zero difference between both methods is within the confidence lines at all time points, suggesting no difference between the measures. Greater data clustering above the upper confidence line versus the lower confidence line suggests a skew in the data toward weights being higher in VHA medical records compared with self-reported results.

## Discussion

While other studies examining agreement between self-reported medical conditions and medical record data exist, to our knowledge, this is the first that incorporated all of the following features: (1) employed two different diagnostic criteria in medical records for case definitions designed to be more sensitive or more specific; (2) employed different temporal restrictions for capture of medical record diagnoses; (3) examined differences in agreement by incorporating medical records from a second care setting to capture diagnoses; and (4) utilized self-reported data from a survey administered repeatedly to participants triennially for up to 20 years. Previous work from the Millennium Cohort Study was only able to examine agreement between three years’ worth of self-report and medical record data from a single data source (i.e., MDR) among participants from the first enrollment panel [1]. This study builds upon and largely corroborates findings from this foundational study by adding over a decade of data from both the MHS and VHA, as well as incorporating 3 additional enrollment panels of service members in the population.

This study observed high levels of negative agreement between self-reported and medical record confirmed diagnoses, with 31 of 39 diagnoses indicating negative agreement > 90%, and only one diagnosis (i.e., depression) indicating negative agreement < 80%. A negative self-report of medical conditions therefore strongly corresponds with a corresponding absence of these conditions in medical records. Positive agreement was much lower, with the highest value observed for hypertension (56.3%), followed by 3 other diagnoses with values > 50% (i.e., migraine headache, depression, PTSD), and 4 others with values between 40 and 50% (i.e., asthma, sleep apnea, hearing loss, tinnitus). The low levels of positive agreement in this study are not surprising, as most conditions examined also have a low prevalence which can impact the level of positive agreement. But in the context of this specific study, the generally low levels of positive agreement may also reflect incorrect self-report of conditions that have not been medically confirmed, self-reported conditions that were not captured due to nonresponse at specific survey waves across time, or failure of the medical record to capture diagnoses due to a lack of proper coding, insufficient frequency of medical care encounters to generate a diagnostic code, incorrect code entry in the medical record, or care received in medical settings not included in these analyses (e.g., outside of VHA or MDR records).

Incorporating MDR records with VHA records provided greater coverage of continuity of care over time, captured a higher prevalence of conditions, and resulted in small increases in positive agreement for most diagnoses (< 10%, except for sinusitis, peptic ulcer, gallstones, pancreatitis, and bladder infection) but little change in overall agreement with self-report. As expected, temporal expansion capturing diagnoses occurring at any time in the medical record as opposed to prior to self-report generally resulted in a higher level of positive agreement for sensitive diagnostic criteria but not for specific criteria. Increasing the time frame for medical record review may have resulted in better capture of valid diagnoses if a delay occurred between the occurrence of a medical condition and entry into the medical record. Positive agreement between medical records and self-reports was lowest among irregular users of VHA health care compared with regular or high frequency users, since more encounters within the health system allow for better capture of diagnoses in the medical record.

The highest agreement of any comparison was seen for self-report of ever smoking. This result is consistent with a systematic review of published studies comparing self-reported smoking with biochemical measures that concluded that self-report is accurate in most studies [25]. Agreement between measures of problem drinking was of a much lower magnitude, likely due to the use of differing tools for self-report in medical records. The AUDIT-C is an extensively validated screen for unhealthy alcohol use, while the PHQ is a screening instrument for detecting the probable presence of multiple mental health conditions [18, 26]. Each tool has its own utility and our results suggest that comparison of the two does not provide equivalent information. Comparison of self-reported and objectively measured height and weight mirrored what has been reported in other populations, with height slightly overestimated and weight underestimated in self-reports [27]. The net effect of this error is to underestimate body mass index, a frequently used proxy measure of body adiposity [28].

Despite the appearance of questions about the presence of medical conditions in national surveys such as those in the U.S. Behavioral Risk Factor Surveillance System, the value of responses to such questions in terms of accurate identification of health conditions of interest is not extensively documented. The available research on self-reported medical diagnoses suggests some limitations to this method. Disparate results can be seen for the same diagnoses, as was shown in research demonstrating high sensitivity (88%) for self-reported diagnosis of rheumatoid arthritis in a systematic review [29], which contrasts with the results of a community-based survey in Norway, where only 19.1% of participants reporting a diagnosis of rheumatoid arthritis were confirmed by medical record review to have this condition [30], and a survey of primary care patients in Boston, where 32% of participants reporting this condition were confirmed as having it from medical record review [31]. Additional difficulties in assessing accuracy of self-reported medical conditions arise from problems intrinsic to the medical record, which has been shown to contain important omissions and inaccuracies [32, 33].

The comparison of existing literature with our results is challenging due to methodologic differences, but some findings can be noted. A meta-analysis that included 22 epidemiological studies of hypertension found that 42.1% of participants diagnosed with this condition reported having it [34]. Similarly, we noted 56.3% positive agreement between medical record diagnosis and self-reported diagnosis. As observed previously in a review of diagnoses resulting in hospitalization, as well as a previous report from the first participants in the Millennium Cohort Study [1, 35], we observed that accuracy of self-reported medical diagnoses varied by condition. While more common conditions were identified similarly between self-reports and VHA records, there were some notable discrepancies between these methods. For instance, mental health (e.g., depression, PTSD) and metabolic conditions (e.g., thyroid conditions, diabetes) were more frequently captured through the medical records, while acute conditions (e.g., bladder infections) were self-reported more often. This may have implications for modes of assessment that better capture cases of conditions, depending on the chronicity and severity of specific medical diagnoses.

This analysis had several limitations. The self-administered survey did not allow for the participant to ask questions to help interpret or clarify any item about which they were unsure. Participants with a condition (e.g., emphysema) may not have responded affirmatively if they recognized it only by a different name (e.g., COPD). Because of the longitudinal nature of this study, some participants may not have responded to all follow-up surveys. Thus, missing data or nonresponse may also contribute to under-documentation of self-reported conditions. Although we compiled extensive lists of codes to capture medical record diagnoses, some may have been overlooked or omitted. We also did not conduct individual medical record reviews due to the large number of participants. Such a review might have detected conditions that were present but either incorrectly coded or not coded (such as those mentioned only in provider notes). Additionally, the medical record review may not have included all sources of care received and may have missed the occurrence of a diagnosis made in a medical setting not included in this analysis. For example, active duty personnel gain automatic eligibility to seek care within the MHS upon entry into the military and all medical encounters among active duty service members should be accounted for in MDR records. However, National Guard and Reserve personnel are only eligible to receive care within the MHS under specific circumstances (e.g., if they have been activated for 30 days or more, if they purchase healthcare plans that allow them to access the MHS) and as such, may routinely seek care outside of the MHS. Likewise, while the use of VHA care is high in our study population, participants may potentially use other sources of care that are not covered by the VA and would not be reflected in the medical records. These potential gaps in documentation of health conditions within the medical records may explain some of the reasons for the low overall positive agreement in this study. As of 2015, 62% of all separated Operations Enduring Freedom, Iraqi Freedom, and New Dawn era veterans had obtained health care from the VA [36]. There are also known differences in sociodemographic and health related characteristics of veterans who use VA healthcare compared with veterans who do not [37, 38], so the population of veterans examined in this study also may not be representative of all veterans within the U.S. Lastly, while the Youden’s J statistic does have benefits in terms of not being prevalence dependent, it also suffers from inherent assumptions of diagnostic accuracy of one measure to evaluate the concordance of another measure. As we cannot assure that either self-report or medical record diagnoses are inherently accurate in this study, this should be considered in the context of our findings and the levels of concordance observed.

## Conclusions

This study demonstrated that negative self-reports of any of the 39 medical conditions surveyed showed a high level of agreement with absence of documentation of the condition in the medical record. Positive agreement was observed at a lower level and varied greatly, depending on the type of condition and related factors such as chronicity. The value and limitations of survey-reported medical conditions should be taken into consideration when using this information for research or planning of clinical care or public health interventions.

## Disclaimer

I am a military service member or employee of the U.S. Government. This work was prepared as part of my official duties. Title 17, U.S.C. §105 provides that copyright protection under this title is not available for any work of the U.S. Government. Title 17, U.S.C. §101 defines a U.S. Government work as work prepared by a military service member or employee of the U.S. Government as part of that person’s official duties. Report No. 24–23 was supported by the Military Operational Medicine Research Program, Defense Health Program, and Department of Veterans Affairs under work unit no. 60002. The views expressed in this article are those of the authors and do not necessarily reflect the official policy or position of the Department of the Navy, Department of Defense, Department of Veterans Affairs, nor the U.S. Government. The study protocol was approved by the Naval Health Research Center Institutional Review Board in compliance with all applicable federal regulations governing the protection of human subjects. Research data were derived from approved Naval Health Research Center Institutional Review Board protocol number NHRC.2000.0007 and VA Puget Sound Institutional Review Board project #1,587,777.

## Supplementary Information

Additional File 1. ICD-9, ICD-10, and CPT codes for the 39 conditions of interest.* Lists all ICD-9, ICD-10, and CPT codes used for case definitions of the 39 conditions of interest, and provides additional data specifications for “kidney failure requiring dialysis.”*

Additional File 2. Sensitivity and specificity of self-report in detecting medical record conditions, by case ascertainment strategy.* Provides the sensitivity and specificity between self-report and medical record diagnoses for the 39 conditions of interest, by different case attainment strategies and time-based criteria.*

Additional File 3. Prevalence and agreement between self-report and medical record conditions, by case ascertainment strategy.* Provides results from supplemental analyses examining the prevalence and agreement for the 39 conditions of interest, by different case attainment strategies and time-based criteria.*

Additional File 4. Prevalence and agreement between self-report and medical record conditions by VHA user frequency, sensitive, at any time criteria.* Provides results from supplemental analyses examining the prevalence and agreement for the 39 conditions of interest, by VHA user frequency.*

Additional File 5. Prevalence and agreement between self-report and medical record diagnoses of depression excluding unspecified depressive disorder, sensitive, at any time criterion.* Provides results from sensitivity analyses examining the prevalence and agreement of depression, excluding ICD codes for Unspecified Depressive Disorder [311 (ICD-9) and F32.9 (ICD-10)].*

## Data Availability

The VHA and DoD datasets used and/or analyzed during the current study are not publicly available due to security protocols and privacy regulations. DoD datasets may be made available on reasonable request by the Naval Health Research Center Institutional Review Board (contact phone +1 619 553 8400) and will require data use agreements to be developed. VA-affiliated researchers can apply to access the VA’s Millennium Cohort Study data at the study website.

## Abbreviations

AUDIT-C: Alcohol Use Disorders Identification Test
COPD: Chronic obstructive pulmonary disease
CPT: Current Procedural Terminology
DMDC: Defense Manpower Data Center
DoD: Department of Defense
ICD: International Classification of Diseases
MDR: Military Health System Data Repository
MHS: Military Health System
PHQ: Patient Health Questionnaire
PTSD: Posttraumatic stress disorder
SD: Standard deviation
USRDS: United States Renal Data System
VHA: Veterans Health Administration
