# Supplementary material for: Comparison of health measures between survey self-reports and electronic health records among Millennium Cohort Study participants receiving Veterans Health Administration care
Source: BMC Med Res Methodol. 2025 Mar 27;25:81. doi: 10.1186/s12874-025-02529-x (PMC11948930; doi:10.1186/s12874-025-02529-x)
Supplement: Supplementary file 1 — Additional File 1. ICD-9, ICD-10, and CPT codes for the 39 conditions of interest. Lists all ICD-9, ICD-10, and CPT codes used for case definitions of the 39 conditions of interest, and provides additional data specifications for “kidney failure requiring dialysis.” [file 12874_2025_2529_MOESM1_ESM.docx]

**Additional File 1.** ICD-9, ICD-10, and CPT codes for the 39 conditions of interest

| **Condition** | **ICD-9** | **ICD-10** | **Personal History** |
| --- | --- | --- | --- |
| Hypertension | 401  401.1  401.9  402  402.01  402.1  402.11  402.9  402.91  403.0  403.00  403.01  403.1  403.1  403.11  403.9  403.9  403.91  404.0  404.00  404.01  404.02  404.03  404.1  404.10  404.11  404.12  404.13  404.9  404.90  404.91  404.92  404.93  405.01  405.09  405.11  405.19  405.91  405.99  437.2  642  642.01  642.02  642.03  642.04  642.1  642.11  642.12  642.13  642.14  642.2  642.21  642.22  642.23  642.24  642.3  642.31  642.32  642.33  642.34  642.4  642.41  642.42  642.43  642.44  642.5  642.51  642.52  642.53  642.54  642.6  642.61  642.62  642.63  642.64  642.7  642.71  642.72  642.73  642.74  642.9  642.91  642.92  642.93  642.94  997.91 | H35.031  H35.032  H35.033  H35.039  I10.0  I11.0  I11.9  I12.0  I12.9  I13.0  I13.10  I13.11  I13.2  I15.0  I15.1  I15.2  I15.8  I15.9  I16.0  I16.1  I16.9  I67.4  N26.2  O10.011  O10.012  O10.013  O10.019  O10.02  O10.03  O10.111  O10.112  O10.113  O10.119  O10.12  O10.13  O10.211  O10.212  O10.213  O10.219  O10.22  O10.23  O10.311  O10.312  O10.313  O10.319  O10.32  O10.33  O10.411  O10.412  O10.413  O10.419  O10.42  O10.43  O10.911  O10.912  O10.913  O10.919  O10.92  O10.93  O11.1  O11.2  O11.3  O11.4  O11.5  O11.9  O13.1  O13.2  O13.3  O13.4  O13.5  O13.9  O16.1  O16.2  O16.3  O16.4  O16.5  O16.9  P00.0  T46.5X6D | 7101  7007  7334  7509  8101 |
| Significant hearing loss | 388.01  388.02  388.12  388.2  389  389.01  389.02  389.03  389.04  389.05  389.06  389.08  389.1  389.11  389.12  389.13  389.14  389.15  389.16  389.17  389.18  389.2  389.2  389.21  389.22  389.7  389.8  389.9  V41.2  V53.2 | H80.00  H80.01  H80.02  H80.03  H80.10  H80.11  H80.12  H80.13  H80.20  H80.21  H80.22  H80.23  H80.80  H80.81  H80.82  H80.83  H80.90  H80.91  H80.92  H80.93  H83.3X1  H83.3X2  H83.3X3  H83.3X9  H90  H91.x  H93.0x  H94.0x  Z46.1 | 6100–6110  6250–6258  6277–6297  6299  6657  8861 |
| Tinnitus | 388.3  388.31  388.32 | H93.11  H93.12  H93.13  H93.19  H93.A1  H93.A2  H93.A3  H93.A9 | 5682  6260  6205 (Meniere’s)  6288 |
| Chronic bronchitis | 490  491  491.1  491.2x  491.8  491.9 | J40  J41.0  J41.1  J41.8  J42  J44 | 6600  6603  6699 |
| Sleep apnea | 327.2  327.21  327.22  327.23  327.24  327.25  327.26  327.27  327.29  780.5  780.51  780.53  780.57 | G47.30  G47.31  G47.33  G47.34  G47.35  G47.36  G47.37  G47.39 | 6699  6847 |
| Rheumatoid arthritis | 714.0  714.1  714.2  714.3x  714.4  714.81 | M05.xxx  M06.xxx  M08.0x  M12.0x | 5002 |
| Thyroid condition other than cancer | 240.0  240.9  241.0  241.1  241.9  242.x  244.x  245.x  246.x  648.14 | E01  E02  E03.x  E04.x  E05.x  E06.x  E07.x | 7008  7900  7903  7901  7902 |
| Cancer | 140.x–208.x  209.0x  209.1x  209.2x  209.3x  230.x–234.x | C00.x–C80.x  C7A.x  C7B.x  C81.x–C96.x  D00.x–D09.x | 5012  5301  5327  5329  6014  6208  6819  7123  7343  7703  7709  7715  7833  7343  7528  7627  7630  7805  7914  7918  8002  8021  8510  8540  9918 |
| Chronic fatigue syndrome | 780.71 | R53.82 | 6354 |
| Diabetes or sugar diabetes | 249.xx, 250.xx, 362.0x, 366.41, 648.0x, 648.8x, V58.67, V45.85, 996.57, 357.2 | E08.xx, E09.xx, E10.xx, E11.xx, E13.xx, O24.xx, Z79.4, Z86.31, Z86.32, T85.6x (complications of insulin pump), T85.72XA, T85.72XD, T85.72XS, Z46.81, Z46.41, E93.23 | 7913 |
| Ulcerative colitis or proctitis | 556  556.x | K51.xx | 7323  7399 |
| Manic-depressive disorder | 296.x | F30  F31 | 9206  9432 |
| Hepatitis C | 070.41  070.44  070.51  070.54  070.70  070.71  V02.62 | B17.1x  B17.1  B17.10  B17.11  B18.2  B19.2  B19.20  B19.21 | 7354  7599 |
| Coronary heart disease | 410.x, 411.x, 411.8, 411.81, 411.89, 412.0, 413.x, 414.x, 996.03  429.71  429.79  V45.81  V45.82 | I20–I25  T82.21x | 7017  7005  7006 |
| Emphysema | 491.2x  492.x  496  506.4 | J43  J44 | 6603  6604 |
| Lupus | 710.0  695.4  373.34 | M32.x  L93.2  L93.0  L93.1  H01.12x | 7809  6350 |
| Multiple sclerosis | 340 | G35 | 8018 |
| Crohn’s disease | 555 | K50  M07.6 (enteropathic arthropathy) | N/A |
| Schizophrenia or psychosis | 295  297  298 | F20–F25  F28  F29 | 9201–9205  9211  9299 |
| Kidney failure requiring dialysis | See following pages for specifics of condition |  | 7502 |
| Cirrhosis | 571.2, 571.5, 571.6 | K70.3, K70.31 K71.7, K74.x | 7312 |
| Sinusitis | 461.x  473.x | J01.x  J32.x | 6510–6514  6599 |
| Migraine | 339.0x  346.x | G43.x  G44.0x | 8100 |
| Depression | 296.2x  296.3x  296.82  309.0  309.1  309.28  311 | F32.x (F32.9)  F33.x  F43.21  F43.23 | 9207  9209  9405  9434 |
| Bladder infection | 595.x, 601.3, 098.11, 098.31, 016.1x, 032.84 | N30.x, N41.3, A54.01, A18.12 | 5698  7514 (tuberculosis)  7512  7513  7599 |
| Asthma | 493.x  519.11 | J45.x  J98.01 | 6602  6699 |
| Any other heart condition | 391.x, 392.0, 393, 398.xx, 420.xx–429.xx | I01.x  I02.0  102.9  I05.x  I06.x  I07.x  I08.0  I09.x  I30.x  I33  133.9  I40.x  I31.x  I34.x  I35.x  I36.x  I37.x  I38  I39  I42.x  I43  I44.x  I45.x  I46.x  I47.x  I48.x  I49.x  I50.x  I51.81  I51.9 | 7000  7004  7008  7010  7011  7012  7013  7014  7015  7016  7018  7019  7020  7099  8870  9110  9111  9501 |
| Anemia | 280.x–285.x | D50.x–D53.x  D55.x–D59.x  D63.x  D64.81  D46.89  D64.x  D64.9 | 7714 (sickle cell)  7716  7700  7701  7720  7722  7723  8525 |
| Stomach, duodenal, or peptic ulcer | 531.x–534.x | K25.x–K28.x | 6305  7302  7304  7305  7306 |
| Angina (chest pain) | 411.1  413.x  413.0  413.1  413.9 | I20.x  125.1x  I25.7.x | 7005  7006  7017  7099 |
| Neuropathy | 354.x–357.x  357.2  250.60  250.61  337.0  337.00  337.01  337.09  337.1 | G60.x–G65.x  G90.9  G90.09  G99.0  M05.5xx  M34.83 | 7535  8205–8799 (excluding 8540 and 8520) |
| Gallstones | 560.31  574.xx | K80.xx  K91.86  K56.3 | 7315 |
| PTSD | 309.81 | F43.1x | 9400  9411 |
| Any other hepatitis | 571.1  571.41  571.42  571.40  571.49  070.9  070.42  070.0  070.43  070.1  070.6  571.1  573.1  573.2  573.3 | B15.0  B15.9  B17.10  B17.11  B17.2  B17.8  B17.9  B18.8  B18.9  B19.0  B19.9  K73.x | 7345 |
| Hepatitis B | 070.2x, 070.3x, V02.61 | B16.x, B17.0, B18, B18.1, B19.10, B19.11 | 7599 |
| Seizures | 345.x (not 345.6)  649.4x  780.3x | F44.5, G40.0xx, R56.xx | 8900 to 8999 |
| Heart attack | 410.xx, 411.0, 412, 429.71, 429.79 | I21.xx, I22.x, I23.x, I24.1, I25.2 | 7006 |
| Pancreatitis | 072.3  577.x | B25.2, B26.3, K85.xx, K86.0, K86.1 | 7347 |
| Stroke | 433.01  433.11  434.01  434.11  434.91  430  431  432.9  435.x  436  437.9  438.x  997.02  435.x | I60.x  I61.x  I62.9  I63.x  I64.x  166.x  I67.8x  I67.9  I69.1x  I69.2x  I69.3x  I97.811  I97.821  G45.x  G46.x | 8007  8008  8009 |

*Note*: 357.2, 250.60, and 250.61 are assigned to diabetes and neuropathy; 411.1 and 413.* are assigned to angina and coronary heart disease; and 491.2* is assigned to chronic bronchitis and emphysema.
ICD-9 and -10, *International Classification of Diseases*, Ninth and Tenth Revisions; CPT, Current Procedural Terminology; PTSD, posttraumatic stress disorder.

**Data Specifications for Kidney Failure Requiring Dialysis**

1. ICD-9-CM or ICD-10-CM diagnosis codes for dialysis

| **Diagnosis** | **ICD-9 diagnosis code** | **ICD-10 diagnosis code** |
| --- | --- | --- |
| Dialysis | V561, V562, V568  V4511–V4512 | Z4901–Z4902  Z4931–Z4932  Z992, Z9115 (noncompliance with renal dialysis) |
| Post-transplant | V420  99681 | Z940  Z4822  T8610–T8613  T8619 |

*Note*: The ICD-9 and ICD-10 diagnosis codes for dialysis are sourced from **the Geriatric & Extended Care Data & Analysis Center under the Department of Veterans Affairs Office of** Geriatrics and Extended Care.
ICD-9 and -10-CM, *International Classification of Diseases*, Ninth and Tenth Revisions, Clinical Modification.

1. ICD-9-PCS or ICD-10-PCS procedure codes for dialysis

| **Procedure** | **ICD-9 procedure code** | **ICD-10 procedure code** |
| --- | --- | --- |
| Creation, revision and removal of arteriovenous fistula or vessel-to-vessel cannula for dialysis | 3927, 3942, 3943, 3993 |  |
| Hemodialysis | 3995 | 5A1D00Z, 5A1D60Z, 5A1D70Z, 5A1D80Z, 5A1D90Z |
| Peritoneal dialysis | 5498 | 3E1M39Z |
| Kidney transplant | 5569 | 0TY00Z0–0TY00Z2  0TY10Z0–0TY10Z2 |

# *Note*: The ICD-9 and ICD-10 procedure codes for dialysis are sourced from the Clinical Classifications Software, a tool developed as part of the Healthcare Cost and Utilization Project, a federal–state–industry partnership sponsored by the Agency for Healthcare Research and Quality. ICD-9 and -10-PCS, *International Classification of Diseases*, Ninth and Tenth Revisions, Procedure Coding System.

1. CPT codes for dialysis

| 4052F, 4053F, G0321–G0327, 90918–90925, 90935, 90937, 90940, 90945, 90947, 90989, 90993, 90997, 90999 |
| --- |

*Note*: The Current Procedural Terminology codes for dialysis are sourced from **the Geriatric & Extended Care Data & Analysis Center under the Department of Veterans Affairs Office of** Geriatrics and Extended Care.
