# Supplementary material for: Comparison of health measures between survey self-reports and electronic health records among Millennium Cohort Study participants receiving Veterans Health Administration care
Source: BMC Med Res Methodol. 2025 Mar 27;25:81. doi: 10.1186/s12874-025-02529-x (PMC11948930; doi:10.1186/s12874-025-02529-x)
Supplement: Supplementary file 2 — Additional File 2. Sensitivity and specificity of self-report in detecting medical record conditions, by case ascertainment strategy. Provides the sensitivity and specificity between self-report and medical record diagnoses for the 39 conditions of interest, by different case attainment strategies and time-based criteria. [file 12874_2025_2529_MOESM2_ESM.docx]

**Additional File 2.** Sensitivity and specificity of self-report in detecting medical record conditions, by case ascertainment strategy

| **Condition** | **Sensitive, at any time** | |  | **Specific, at any time** | |  | **Sensitive, prior to survey date** | |  | **Specific, prior to survey date** | |
| --- | --- | --- | --- | --- | --- | --- | --- | --- | --- | --- | --- |
|  | Sensitivity | Specificity |  | Sensitivity | Specificity |  | Sensitivity | Specificity |  | Sensitivity | Specificity |
| **Diseases & Disorders of the Nervous System** | | | | | | | | | | | |
| Multiple sclerosis | 45.2% | 99.4% |  | 55.5% | 99.4% |  | 62.5% | 99.3% |  | 80.6% | 99.3% |
| Migraine headaches | 61.4% | 84.0% |  | 65.7% | 82.5% |  | 77.5% | 78.0% |  | 83.0% | 77.8% |
| Neuropathy | 25.0% | 94.3% |  | 29.2% | 93.8% |  | 26.0% | 93.4% |  | 30.3% | 93.2% |
| Seizures | 28.6% | 98.9% |  | 37.1% | 98.8% |  | 43.1% | 98.7% |  | 60.7% | 98.6% |
| Stroke | 16.5% | 99.3% |  | 22.5% | 99.3% |  | 32.0% | 99.2% |  | 46.9% | 99.2% |
| Sleep apnea | 37.2% | 91.8% |  | 40.3% | 90.9% |  | 65.6% | 87.3% |  | 73.3% | 87.2% |
| **Diseases & Disorders of the Sense Organs** | | | | | | | | | | | |
| Significant hearing loss | 43.2% | 89.4% |  | 55.2% | 87.5% |  | 37.0% | 84.3% |  | 50.8% | 84.0% |
| Tinnitus | 46.0% | 84.1% |  | 53.4% | 81.1% |  | 71.5% | 81.9% |  | 78.5% | 80.2% |
| **Diseases & Disorders of the Respiratory System** | | | | | | | | | | | |
| Asthma | 50.2% | 94.5% |  | 57.7% | 93.7% |  | 60.6% | 91.7% |  | 71.7% | 91.5% |
| Chronic bronchitis | 20.6% | 94.2% |  | 30.1% | 93.9% |  | 17.3% | 93.8% |  | 27.3% | 93.7% |
| Emphysema | 8.6% | 99.3% |  | 14.0% | 99.2% |  | 11.8% | 99.2% |  | 20.2% | 99.1% |
| Sinusitis | 38.0% | 82.7% |  | 47.2% | 81.7% |  | 33.5% | 80.4% |  | 43.2% | 80.3% |
| **Diseases & Disorders of the Circulatory System** | | | | | | | | | | | |
| Hypertension | 54.2% | 88.3% |  | 59.6% | 87.1% |  | 69.2% | 81.0% |  | 75.9% | 80.9% |
| Coronary heart disease | 27.1% | 98.9% |  | 41.9% | 98.8% |  | 28.6% | 98.7% |  | 46.4% | 98.6% |
| Angina | 28.4% | 94.3% |  | 35.1% | 94.1% |  | 38.7% | 93.8% |  | 54.4% | 93.8% |
| Heart attack | 28.2% | 99.0% |  | 31.6% | 98.9% |  | 48.3% | 98.8% |  | 62.9% | 98.8% |
| Any other heart condition | 22.3% | 93.6% |  | 30.1% | 93.2% |  | 20.8% | 92.5% |  | 30.8% | 92.4% |
| **Diseases & Disorders of the Digestive System** | | | | | | | | | | | |
| Stomach, duodenal, or peptic ulcer | 36.3% | 95.0% |  | 36.9% | 94.8% |  | 45.9% | 94.8% |  | 52.5% | 94.7% |
| Ulcerative colitis or proctitis | 33.9% | 98.8% |  | 50.3% | 98.8% |  | 62.6% | 98.7% |  | 76.3% | 98.7% |
| Crohn's disease | 39.1% | 99.5% |  | 49.9% | 99.5% |  | 62.9% | 99.4% |  | 74.5% | 99.4% |
| **Diseases & Disorders of the Hepatobiliary System & Pancreas** | | | | | | | | | | | |
| Hepatitis B | 38.7% | 99.1% |  | 50.3% | 99.1% |  | 33.0% | 99.0% |  | 51.0% | 99.0% |
| Hepatitis C | 29.7% | 99.4% |  | 40.3% | 99.4% |  | 38.6% | 99.3% |  | 62.9% | 99.3% |
| Any other hepatitis | 10.8% | 98.8% |  | 12.2% | 98.8% |  | 11.4% | 98.8% |  | 14.6% | 98.8% |
| Cirrhosis | 9.9% | 99.4% |  | 12.5% | 99.4% |  | 37.7% | 99.4% |  | 48.9% | 99.4% |
| Gallstones | 22.3% | 96.8% |  | 24.6% | 96.8% |  | 61.9% | 96.8% |  | 65.3% | 96.7% |
| Pancreatitis | 20.6% | 99.1% |  | 22.2% | 99.1% |  | 50.6% | 99.1% |  | 61.2% | 99.1% |
| **Diseases & Disorders of the Musculoskeletal System & Connective Tissue** | | | | | | | | | | | |
| Rheumatoid arthritis | 39.5% | 94.6% |  | 46.5% | 94.5% |  | 57.0% | 94.3% |  | 75.6% | 94.3% |
| Lupus | 41.8% | 99.4% |  | 48.0% | 99.4% |  | 62.5% | 99.3% |  | 72.3% | 99.3% |
| **Endocrine, Nutritional & Metabolic Diseases & Disorders** | | | | | | | | | | | |
| Thyroid condition other than cancer | 38.6% | 97.6% |  | 44.6% | 97.3% |  | 57.4% | 95.8% |  | 64.9% | 95.8% |
| Diabetes or sugar diabetes | 32.9% | 98.0% |  | 38.2% | 97.9% |  | 67.3% | 96.8% |  | 76.1% | 96.8% |
| **Diseases & Disorders of the Kidney & Urinary Tract** | | | | | | | | | | | |
| Bladder infection | 37.4% | 90.3% |  | 46.1% | 90.0% |  | 30.9% | 89.9% |  | 40.5% | 89.9% |
| Kidney failure requiring dialysis* | 19.3% | 99.5% |  | 19.3% | 99.5% |  | 47.9% | 99.5% |  | 47.9% | 99.5% |
| **Disorders of Blood and Blood Forming Organs** | | | | | | | | | | | |
| Anemia | 27.7% | 93.9% |  | 31.1% | 93.4% |  | 31.3% | 92.5% |  | 35.7% | 92.5% |
| **Mental and Behavioral Disorders** | | | | | | | | | | | |
| Manic depressive disorder | 16.5% | 98.2% |  | 19.9% | 98.1% |  | 21.3% | 97.7% |  | 27.2% | 97.6% |
| Schizophrenia or psychosis | 13.1% | 99.3% |  | 17.1% | 99.2% |  | 25.5% | 99.2% |  | 35.2% | 99.2% |
| Depression | 46.6% | 86.7% |  | 49.3% | 85.1% |  | 63.7% | 77.8% |  | 67.5% | 77.6% |
| Posttraumatic stress disorder | 42.3% | 93.7% |  | 45.0% | 92.8% |  | 71.4% | 87.7% |  | 75.4% | 87.5% |
| **Other Conditions** | | | | | | | | | | | |
| Cancer | 33.9% | 97.5% |  | 40.1% | 97.0% |  | 50.7% | 96.4% |  | 66.8% | 96.4% |
| Chronic fatigue syndrome | 14.9% | 96.8% |  | 22.9% | 96.8% |  | 32.5% | 96.7% |  | 47.3% | 96.7% |

*Note*: Sensitive case ascertainment is defined as any diagnostic code appearing in any Veterans Health Administration (VHA) record; specific case ascertainment is defined as two codes corresponding to the same diagnosis from outpatient encounters or one diagnostic code from an inpatient encounter.
^*^Sensitive and specific case ascertainment for kidney failure requiring dialysis are equivalent and defined as one code corresponding to any encounter.
