# Supplementary material for: Comparison of health measures between survey self-reports and electronic health records among Millennium Cohort Study participants receiving Veterans Health Administration care
Source: BMC Med Res Methodol. 2025 Mar 27;25:81. doi: 10.1186/s12874-025-02529-x (PMC11948930; doi:10.1186/s12874-025-02529-x)
Supplement: Supplementary file 3 — Additional File 3. Prevalence and agreement between self-report and medical record conditions, by case ascertainment strategy. Provides results from supplemental analyses examining the prevalence and agreement for the 39 conditions of interest, by different case attainment strategies and time-based criteria. [file 12874_2025_2529_MOESM3_ESM.docx]

**Additional File 3.** Prevalence and agreement between self-report and medical record conditions, by case ascertainment strategy

|  | **Sensitive, prior to survey date** | | | |  | **Specific, at any time** | | | |  | **Specific, prior to survey date** | | | |
| --- | --- | --- | --- | --- | --- | --- | --- | --- | --- | --- | --- | --- | --- | --- |
| **Condition** | n (%) | Positive agreement | Negative agreement | Youden’s *J* |  | n (%) | Positive agreement | Negative agreement | Youden’s *J* |  | n (%) | Positive agreement | Negative agreement | Youden’s *J* |
| **Diseases & Disorders of the Nervous System** | | | | | | | | | | | | | | |
| Multiple sclerosis | 144 (0.1) | 17.5% | 99.6% | 0.62 |  | 357 (0.3) | 31.8% | 99.6% | 0.55 |  | 108 (0.1) | 17.5% | 99.6% | 0.80 |
| Migraine headaches | 2885 (2.5) | 14.9% | 87.3% | 0.55 |  | 14,264 (12.3) | 45.2% | 88.1% | 0.48 |  | 2261 (1.9) | 12.7% | 87.3% | 0.61 |
| Neuropathy | 4592 (4.0) | 18.2% | 95.1% | 0.19 |  | 6059 (5.2) | 24.2% | 94.9% | 0.23 |  | 2934 (2.5) | 15.5% | 95.6% | 0.24 |
| Seizures | 721(0.6) | 24.0% | 99.1% | 0.42 |  | 1432 (1.2) | 32.2% | 99.0% | 0.36 |  | 443 (0.4) | 23.3% | 99.2% | 0.59 |
| Stroke | 482 (0.4) | 19.6% | 99.5% | 0.31 |  | 1065 (0.9) | 22.3% | 99.3% | 0.22 |  | 290 (0.2) | 19.7% | 99.5% | 0.46 |
| Sleep apnea | 6973 (6.0) | 36.1% | 92.2% | 0.53 |  | 24,958 (21.5) | 46.4% | 87.7% | 0.31 |  | 5827 (5.0) | 35.3% | 92.5% | 0.61 |
| **Diseases & Disorders of the Sense Organs** | | | | | | | | | | | | | | |
| Significant hearing loss | 8044 (6.9) | 21.2% | 89.2% | 0.21 |  | 12,723 (10.9) | 42.9% | 90.6% | 0.43 |  | 3994 (3.4) | 16.9% | 90.4% | 0.35 |
| Tinnitus | 8599 (7.4) | 35.9% | 88.9% | 0.53 |  | 10,553 (9.1) | 31.1% | 87.3% | 0.34 |  | 4396 (3.8) | 23.0% | 88.6% | 0.59 |
| **Diseases & Disorders of the Respiratory System** | | | | | | | | | | | | | | |
| Asthma | 2193 (1.9) | 20.4% | 95.3% | 0.52 |  | 6921 (6.0) | 45.0% | 95.4% | 0.51 |  | 1589 (1.4) | 18.3% | 95.4% | 0.63 |
| Chronic bronchitis | 1767 (1.5) | 6.6% | 96.2% | 0.11 |  | 1303 (1.1) | 9.0% | 96.4% | 0.24 |  | 501 (0.4) | 3.4% | 96.6% | 0.21 |
| Emphysema | 1133 (1.0) | 11.9% | 99.1% | 0.11 |  | 1737 (1.5) | 17.1% | 99.0% | 0.13 |  | 593 (0.5) | 14.1% | 99.4% | 0.19 |
| Sinusitis | 3831 (3.3) | 9.5% | 88.0% | 0.14 |  | 7069 (6.1) | 21.9% | 88.3% | 0.29 |  | 1956 (1.7) | 6.7% | 88.6% | 0.24 |
| **Diseases & Disorders of the Circulatory System** | | | | | | | | | | | | | | |
| Hypertension | 6189 (5.3) | 27.3% | 88.7% | 0.50 |  | 21,901 (18.8) | 55.4% | 88.7% | 0.47 |  | 5275 (4.5) | 26.3% | 88.9% | 0.57 |
| Coronary heart disease | 1110 (1.0) | 21.3% | 99.0% | 0.27 |  | 1274 (1.1) | 34.0% | 99.1% | 0.41 |  | 593 (0.5) | 22.4% | 99.2% | 0.45 |
| Angina | 310 (0.3) | 3.2% | 96.7% | 0.33 |  | 1592 (1.4) | 12.6% | 96.5% | 0.29 |  | 158 (0.1) | 2.3% | 96.8% | 0.48 |
| Heart attack | 176 (0.2) | 10.6% | 99.4% | 0.47 |  | 580 (0.5) | 18.3% | 99.3% | 0.30 |  | 116 (0.1) | 9.5% | 99.4% | 0.62 |
| Any other heart condition | 3345 (2.9) | 11.1% | 94.9% | 0.13 |  | 5492 (4.7) | 22.5% | 94.8% | 0.23 |  | 1739 (1.5) | 9.8% | 95.6% | 0.23 |
| **Diseases & Disorders of the Digestive System** | | | | | | | | | | | | | | |
| Stomach, duodenal, or peptic ulcer | 294 (0.3) | 4.1% | 97.2% | 0.41 |  | 590 (0.5) | 6.4% | 97.2% | 0.32 |  | 141 (0.1) | 2.3% | 97.3% | 0.47 |
| Ulcerative colitis or proctitis | 131 (0.1) | 9.4% | 99.3% | 0.61 |  | 477 (0.4) | 22.9% | 99.3% | 0.49 |  | 93 (0.1) | 8.3% | 99.3% | 0.75 |
| Crohn’s disease | 124 (0.1) | 16.6% | 99.7% | 0.62 |  | 367 (0.3) | 31.0% | 99.6% | 0.49 |  | 98 (0.1) | 16.0% | 99.7% | 0.74 |
| **Diseases & Disorders of the Hepatobiliary System & Pancreas** | | | | | | | | | | | | | | |
| Hepatitis B | 88 (0.1) | 4.7% | 99.5% | 0.32 |  | 171 (0.1) | 13.1% | 99.5% | 0.49 |  | 51 (0.04) | 4.3% | 99.5% | 0.50 |
| Hepatitis C | 254 (0.2) | 16.4% | 99.6% | 0.38 |  | 524 (0.5) | 28.8% | 99.5% | 0.40 |  | 143 (0.1) | 16.6% | 99.6% | 0.62 |
| Any other hepatitis | 245 (0.2) | 3.4% | 99.3% | 0.10 |  | 263 (0.2) | 3.8% | 99.3% | 0.11 |  | 123 (0.1) | 2.3% | 99.4% | 0.13 |
| Cirrhosis | 69 (0.1) | 6.6% | 99.7% | 0.37 |  | 312 (0.3) | 7.6% | 99.6% | 0.12 |  | 47 (0.04) | 6.0% | 99.7% | 0.48 |
| Gallstones | 488 (0.4) | 13.4% | 98.3% | 0.59 |  | 1327 (1.1) | 12.2% | 97.9% | 0.21 |  | 372 (0.3) | 11.0% | 98.3% | 0.62 |
| Pancreatitis | 166 (0.1) | 12.7% | 99.5% | 0.50 |  | 567 (0.5) | 14.6% | 99.4% | 0.21 |  | 116 (0.1) | 11.1% | 99.5% | 0.60 |
| **Diseases & Disorders of the Musculoskeletal System & Connective Tissue** | | | | | | | | | | | | | | |
| Rheumatoid arthritis | 193 (0.2) | 3.2% | 97.1% | 0.51 |  | 660 (0.6) | 8.4% | 97.0% | 0.41 |  | 119 (0.1) | 2.6% | 97.1% | 0.70 |
| Lupus | 80 (0.1) | 11.2% | 99.7% | 0.62 |  | 300 (0.3) | 25.0% | 99.6% | 0.47 |  | 65 (0.1) | 10.7% | 99.7% | 0.72 |
| **Endocrine, Nutritional & Metabolic Diseases & Disorders** | | | | | | | | | | | | | | |
| Thyroid condition other than cancer | 2182 (1.9) | 30.7% | 97.5% | 0.53 |  | 6764 (5.8) | 47.3% | 96.9% | 0.42 |  | 1813 (1.6) | 30.1% | 97.6% | 0.61 |
| Diabetes or sugar diabetes | 2318 (2.0) | 41.7% | 98.1% | 0.64 |  | 7429 (6.4) | 45.1% | 96.9% | 0.36 |  | 1990 (1.7) | 42.4% | 98.2% | 0.73 |
| **Diseases & Disorders of the Kidney & Urinary Tract** | | | | | | | | | | | | | | |
| Bladder infection | 246 (0.2) | 1.3% | 94.6% | 0.21 |  | 531 (0.5) | 4.0% | 94.6% | 0.36 |  | 74 (0.1) | 0.5% | 94.6% | 0.30 |
| Kidney failure requiring dialysis^*^ | 71 (0.1) | 10.3% | 99.7% | 0.47 |  | 249 (0.2) | 11.5% | 99.7% | 0.19 |  | 71 (0.1) | 10.3% | 99.7% | 0.47 |
| **Disorders of Blood and Blood Forming Organs** | | | | | | | | | | | | | | |
| Anemia | 2363 (2.0) | 12.7% | 95.4% | 0.24 |  | 6560 (5.6) | 25.8% | 94.6% | 0.24 |  | 1735 (1.5) | 11.3% | 95.6% | 0.28 |
| **Mental and Behavioral Disorders** | | | | | | | | | | | | | | |
| Manic depressive disorder | 2902 (2.5) | 20.0% | 97.8% | 0.19 |  | 5697 (4.9) | 25.2% | 97.0% | 0.18 |  | 2073 (1.8) | 21.0% | 98.1% | 0.25 |
| Schizophrenia or psychosis | 737 (0.6) | 19.9% | 99.3% | 0.25 |  | 1670 (1.4) | 20.2% | 99.0% | 0.16 |  | 505 (0.4) | 21.5% | 99.4% | 0.34 |
| Depression | 11,980 (10.3) | 35.7% | 85.5% | 0.42 |  | 39,017 (33.6) | 55.2% | 80.8% | 0.34 |  | 10,556 (9.1) | 34.5% | 85.8% | 0.45 |
| Posttraumatic stress disorder | 9603 (8.3) | 46.4% | 92.2% | 0.59 |  | 30,787 (26.5) | 54.6% | 87.3% | 0.38 |  | 8677 (7.5) | 45.7% | 92.4% | 0.63 |
| **Other Conditions** | | | | | | | | | | | | | | |
| Cancer | 1424 (1.2) | 23.2% | 97.9% | 0.47 |  | 3531 (3.0) | 34.0% | 97.5% | 0.37 |  | 964 (0.8) | 22.3% | 98.0% | 0.63 |
| Chronic fatigue syndrome | 169 (0.2) | 2.7% | 98.3% | 0.29 |  | 595 (0.5) | 6.1% | 98.2% | 0.20 |  | 74 (0.1) | 1.8% | 98.3% | 0.44 |

*Note*: Sensitive case ascertainment is defined as any diagnostic code appearing in any Veterans Health Administration (VHA) record; specific case ascertainment is defined as two codes corresponding to the same diagnosis from outpatient encounters or one diagnostic code from an inpatient encounter; sensitive, at any time criteria are used in main analyses (Table 2).
^*^Sensitive and specific case ascertainment for kidney failure requiring dialysis are equivalent and defined as one code corresponding to any encounter.
