# Supplementary material for: Comparison of health measures between survey self-reports and electronic health records among Millennium Cohort Study participants receiving Veterans Health Administration care
Source: BMC Med Res Methodol. 2025 Mar 27;25:81. doi: 10.1186/s12874-025-02529-x (PMC11948930; doi:10.1186/s12874-025-02529-x)
Supplement: Supplementary file 5 — Additional File 5. Prevalence and agreement between self-report and medical record diagnoses of depression excluding unspecified depressive disorder, sensitive, at any time criterion. Provides results from sensitivity analyses examining the prevalence and agreement of depression, excluding ICD codes for Unspecified Depressive Disorder [311 (ICD-9) and F32.9 (ICD-10)]. [file 12874_2025_2529_MOESM5_ESM.docx]

**Additional File 5.** Prevalence and agreement between self-report and medical record diagnoses of depression excluding unspecified depressive disorder, sensitive, at any time criterion (N=116,288)

|  | **Self-report** |  | **VHA records only** | | | |  | **Combined VHA-MDR records** | | | |
| --- | --- | --- | --- | --- | --- | --- | --- | --- | --- | --- | --- |
| **Condition** | n (%) |  | n (%) | Positive agreement | Negative agreement | Youden’s *J* |  | n (%) | Positive agreement | Negative agreement | Youden’s *J* |
| Depression | 30,780 (26.5) |  | 37,959 (32.6) | 52.5% | 80.1% | 0.31 |  | 50,650 (43.6) | 56.9% | 76.8% | 0.34 |

*Note.* Excluded codes include 311 (ICD-9) and F32.9 (ICD-10).

MDR, Military Health System Data Repository; VHA, Veterans Health Administration.
